# Expression profiling of ileal mucosa in asthma reveals upregulation of innate immunity and genes characteristic of Paneth and goblet cells

**DOI:** 10.1186/s13223-021-00584-9

**Published:** 2021-07-31

**Authors:** Jan K. Nowak, Marzena Dworacka, Nazgul Gubaj, Arystan Dossimov, Zhumabek Dossimov, Jarosław Walkowiak

**Affiliations:** 1 Department of Pediatric Gastroenterology and Metabolic Diseases, Poznan University of Medical Sciences, ul. Szpitalna 27/33, 60-572 Poznan, Poland; 2 Department of Pharmacology, Poznan University of Medical Sciences, Poznan, Poland; 3 Department of Pediatric Diseases No. 2, West Kazakhstan Marat Ospanov Medical University, Aktobe, Kazakhstan

**Keywords:** Crohn, Inflammatory bowel disease, Mucin, Ileum, Airway

## Abstract

**Background:**

The expression profiles of the intestinal mucosa have not been comprehensively investigated in asthma. We aimed to explore this in the Correlated Expression and Disease Association Research (CEDAR) patient cohort.

**Methods:**

Differential expression analysis of ileal, transverse colon, and rectal biopsies were supplemented by a comparison of transcriptomes from platelets and leukocytes subsets, including CD4+, CD8+, CD14+, CD15+, and CD19+ cells. Asthma patients (n = 15) and controls (n = 15) had similar age (*p* = 0.967), body mass index (*p* = 0.870), similar numbers of females (80%) and smoking rates (13.3%).

**Results:**

Significant differential expression was found in the ileum alone, and not in any other cell/tissue types. More genes were found to be overexpressed (1,150) than under-expressed (380). The most overexpressed genes included Fc Fragment of IgG Binding Protein (*FCGBP*, logFC = 3.01, pFDR = 0.015), Mucin 2 (*MUC2*, logFC = 2.78, pFDR = 0.015), and Alpha 1B Defensin (*DEFA1B*, logFC = 2.73, pFDR = 0.024). Gene ontology implicated the immune system, including interleukins 4 and 13, as well as antimicrobial peptides in this overexpression. There was concordance of gene over- (*STAT1*, *XBP1*) and underexpression (*NELF*, *RARA*) in asthma and Crohn’s disease ileum when our results were compared to another dataset (p = 3.66 × 10^–7^).

**Conclusion:**

Ileal mucosa in asthma exhibits a specific transcriptomic profile, which includes the overexpression of innate immune genes, mostly characteristic of Paneth and goblet cells, in addition to other changes that may resemble Crohn’s disease.

**Supplementary Information:**

The online version contains supplementary material available at 10.1186/s13223-021-00584-9.

## Background

Asthma is a heterogeneous disease of complex origin that affects over 300 million patients worldwide, making it one of the most common diseases [1]. The chief complaint of shortness of breath is facilitated by airflow obstruction that results from bronchoconstriction and airway remodeling in response to various factors, such as allergens and pollutants. A hallmark of the predominant asthma subtype is the stimulation of the adaptive immune system [2], specifically a Th2-type response, with involvement of interleukin (IL)-4 and IL-13, as well as antigen-specific immunoglobulin (Ig) type E. Type 2 innate immunity typically involves type 2 innate lymphoid cells, especially in older patients. The epithelial compartment can also respond to environmental stimuli with a range of cytokines (including IL-33, IL-25, TSLP, and IL-1alpha) that may trigger and perpetuate airway inflammation. Asthma, which is not dependent on type 2 immunity (and often steroid-resistant), may be related to airway neutrophilia, increased tumor necrosis factor (TNF) alpha, and therefore to Th17 responses. Moreover, there is probably a smaller role for interferons, and systemic inflammation mediated by IL-1β and IL-6 [2]. Recent transcriptomic studies have uncovered how the intricate regulation of the immune system is disturbed in asthma at the single-cell level [3]. Finally it is thought that this multifaceted pathological state is largely dependent on lifestyle, as shown by the increasing occurrence trend over the last 50 years.

Similar to asthma, the incidence of other immune-mediated diseases has increased since the 1970s. These include inflammatory bowel diseases (IBD) that affect 0.5% of the population of developed countries [4]. Analogies have been previously drawn between IBD and asthma [5, 6], the risk of which is increased by approximately 50% in IBD patients [7, 8]. This suggests that airway mucosal pathology could co-occur with intestinal mucosa dysfunction. In fact, there are many more lines of evidence supporting intestinal involvement in asthma separate from IBD epidemiology. Intestinal permeability was shown to be twice as high in asthma patients as in controls or chronic obstructive pulmonary disease [9]. Pathomorphological studies of gastrointestinal mucosa of asthma patients indicated increased activity of mucus-producing cells (as in the bronchi), barrier degeneration, and hyperplasia [10]. Historically, a major gastrointestinal focus in asthma has been helminth infections [11]. However, the center of attention has now shifted to the microbiota, including archaea [12]. Interestingly, the intestinal microflora of asthma patients produces less fatty acids, such as butyrate, propionate, and acetate, which are key for intestinal homeostasis [13]. This dysbiosis likely primes the immune system for asthma development as early as in the first months of life [14]. Accordingly, it has been found that subclinical intestinal inflammation, as measured with calprotectin in infants, associates with future development of asthma [15]. Overall, the evidence is sufficient to implicate the gut in asthma development, however the details of asthma-related intestinal mucosa pathology remain unknown. To bridge this gap, we hypothesized that gene expression profiles of intestinal mucosa differ between patients with asthma and controls.

## Methods

### *Dataset from the CEDAR study by Momozawa *et al*.*

Correlated Expression and Disease Association Research (CEDAR) is a cohort of 323 healthy Europeans recruited by Momozawa et al. in Liège (Belgium) within a colon cancer screening program [16]. Apart from participants without disease, CEDAR also recruited 38 persons with various illnesses, including a sub-population with asthma (n = 15).

Blood samples were obtained from each participant and subpopulations of peripheral blood mononuclear cells were isolated using antibodies against CD4+, CD8+, CD14+, CD15+, and CD19+conjugated to magnetic beads (Miltenyi Biotec, Bergisch Gladbach, Germany). Platelet-rich plasma was centrifuged and subject to CD45+ selection in order to isolate platelets. Finally, biopsies were obtained from three regions of the intestine, namely the ileum, transverse colon, and rectum. Expression profiling was conducted using HT-12 Expression Beadchip (Illumina, San Diego, CA, USA).

### Study analysis

The dataset was accessed at ArrayExpress (E-MTAB-6667). Fifteen controls were selected to optimally match age and sex of patients with asthma, followed by smoking status, and body mass index (BMI).

Raw data were read using *limma* separately for each tissue and/or cell type. After verifying that sufficient intensity was reached in each microarray, the data were normalized using the *neqc* function. Occasional duplicates were not removed. *Limma* was used to conduct differential expression analysis centered on the asthma-control contrast. P-values were corrected using the Benjamini–Hochberg method (false-discovery ratio procedure, FDR). The top 100 overexpressed and underexpressed genes were subject to ontology analysis with (a) Reactome (release 75, Pathway Browser 3.7) and (b) gene set enrichment analysis (GSEA) using biological process ontology from the Broad Institute (MSigDB v7.2). The volcano plot was prepared using *ggplot2* with *ggrepel*.

To check for similarity to the ileal expression profile in Crohn’s disease (CD), asthma samples were compared with a dataset from Vancamelbeke et al. [17] that was accessed via the R2 platform at the Amsterdam Medical Center. CD *vs*. normal ileum was compared using *limma*, with the p_FDR_ threshold set at 0.05. The complete results from both the asthma and CD analyses were intersected and analyzed for overexpression concordance using a McNemar’s test. Whereas the Vancamelbeke et al. dataset available through R2 was collapsed to single-gene resolution, we retained the probe level resolution in the asthma analysis. The top 150 over- and under-expressed genes of asthma and CD samples were intersected to provide the lists of transcripts that contribute to similarity and dissimilarity between asthma and CD ileal samples.

This study exploits existing data and therefore only a post-hoc power calculation could be done. Analyses using *limma* revealed that the study was able to detect 0.70 log-fold change in the asthmatic ileum, depending on the characteristics of expression of individual genes (from as low as 0.40). The number of genes included in the analyses varied between tissue and cell types (with significantly lower expression in platelets), changing the significance threshold and therefore affecting the validity of the power estimate. As this study used published data, we did not require institutional bioethical approval.

## Results

### Patients

Fifteen patients were diagnosed with asthma (and no comorbidities) in the CEDAR cohort. Fifteen controls were selected to optimally match the cases based on age, gender, smoking status and BMI (Table 1). Smoking controls had the same sex and similar age to the asthmatic cases. The characteristics of all patients and CEDAR identifiers are given in Table 2.

**Table 1:** Group characteristics

Characteristic	Asthma (n = 15)	Control (n = 15)	P
Age, years	54.3 ± 16.4	54.4 ± 16.0	0.967
Sex, female	12 (80.0%)	12 (80.0%)	1.0
BMI, kg/m^2^	27.4 ± 5.0	26.8 ± 4.7	0.870
Smoking	3 (13.3%)	3 (13.3%)	1.0

Age and body mass index (BMI) were compared using the Mann–Whitney U test. Sex and smoking status were compared using the Fisher’s exact test

**Table 2:** Characteristics of matched pairs of CEDAR study participants

Patients with asthma					Controls				
Sex	Age, years	BMI, kg/m^2^	Smoking status	CEDAR ID	Sex	Age, years	BMI, kg/m2	Smoking status	CEDAR ID
Female	28	34,63	Non-smoker	IPC386	Female	30	26,95	Non-smoker	IPC102
Female	36	36,33	Smoker	IPC361	Female	36	32,37	Smoker	IPC206
Female	36	26,56	Non-smoker	IPC300	Female	36	33,06	Non-smoker	IPC250
Male	37	30,35	Non-smoker	IPC383	Male	37	28,4	Non-smoker	IPC353
Female	45	19,27	Non-smoker	IPC376	Female	45	17,9	Non-smoker	IPC114
Female	48	23,84	Non-smoker	IPC368	Female	49	21,72	Non-smoker	IPC331
Female	53	31,22	Smoker	IPC072	Female	53	26,75	Smoker	IPC051
Female	54	31,25	Non-smoker	IPC371	Female	55	28,62	Non-smoker	IPC213
Female	55	24,09	Non-smoker	IPC401	Female	55	23,03	Non-smoker	IPC132
Female	58	31,25	Non-smoker	IPC043	Female	58	33,87	Non-smoker	IPC379
Female	60	28,09	Non-smoker	IPC292	Female	60	29,41	Non-smoker	IPC036
Female	72	21,89	Non-smoker	IPC327	Female	71	25,47	Non-smoker	IPC159
Female	73	21,51	Non-smoker	IPC342	Female	73	20,55	Non-smoker	IPC370
Male	78	24,86	Non-smoker	IPC055	Male	78	24,31	Non-smoker	IPC163
Male	81	25,95	Non-smoker	IPC315	Male	80	29,03	Non-smoker	IPC023

### Differential gene expression

**Fig. 1:**
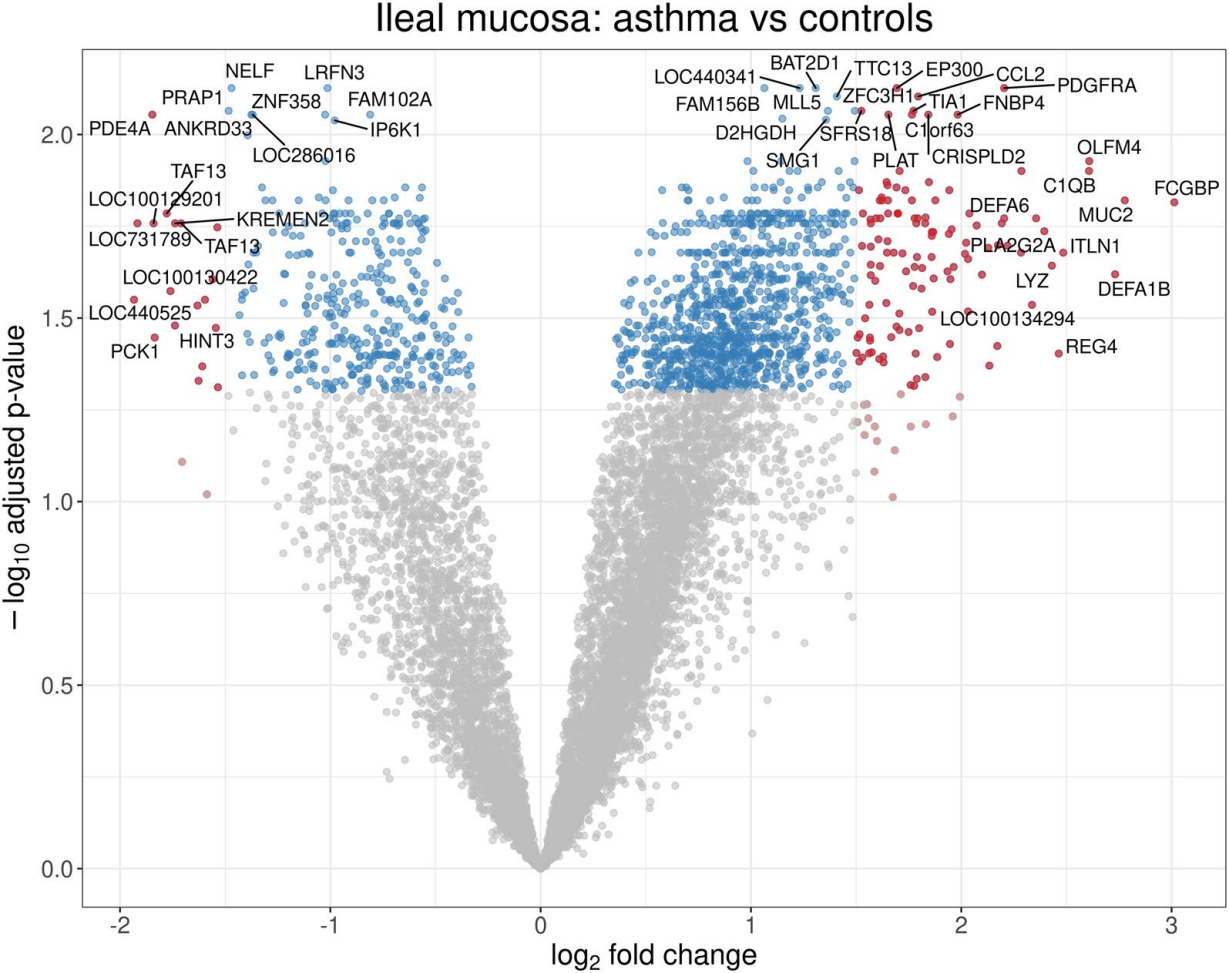
Volcano plot of differential gene expression in ileum of patients with asthma vs matched controls. Significant results are shown in color: red and blue for, respectively, absolute log2 fold change greater or smaller than 1.5. Most of the significant genes are overexpressed, many of which relate to innate (*DEFA1B, DEFA6, ITLN1, LYZ, MUC2*) or adaptive immunity (*FCGBP, C1QB*)

The number of genes with log_2_(expression) > 6 was 10,667. Of these, 1530 were differentially expressed in ileal biopsies from asthma patients compared with controls (Fig. 1). More genes were overexpressed (1150) than underexpressed (380). The number of genes differentially expressed with absolute log_2_ fold change > 1 was 635, of which 520 were overexpressed and 115 underexpressed. The top overexpressed genes included *FCGBP*, *MUC2*, and *DEFA1B* (Table 3). Conversely, *PDE4A*, *PCK1*, and *TAF13* were the most downregulated transcripts (Table 4).

**Table 3:** Top 20 genes differentially expressed in ileal mucosa ranked by absolute log2 fold change (logFC) in asthma vs controls. FDR—false-discovery ratio (Benjamini-Hochberg)

logFC	p_FDR_	Gene	Gene name and function
3.01	0.015	*FCGBP*	Fc Fragment Of IgG Binding Protein—binds IgG Fc and MUC2
2.78	0.015	*MUC2*	Mucin 2, Oligomeric Mucus/Gel-Forming—forms mucus layer protecting the intestinal (and airway) epithelium
2.73	0.024	*DEFA1B*	Defensin Alpha 1B—antimicrobial peptide
2.61	0.012	*OLFM4*	Olfactomedin 4—intestinal stem cell marker, also secreted by neutrophils during the formation of extracellular traps (NETs)
2.61	0.013	*C1QB*	Complement C1q B Chain—the complement bridges innate and adaptive immunity
2.48	0.021	*ITLN1*	Intelectin 1—binds bacterial glycans; adipokine (also known as omentin)
2.46	0.040	*REG4*	Regenerating Family Member 4—lectin-like protein binding manno-oligosaccharides
2.43	0.023	*LYZ*	Lysozyme—cleaves bacterial peptidoglycan
2.39	0.018	*PLA2G2A*	Phospholipase A2 Group IIA—phospholipase degrading bacterial membrane phospholipids; promotes Paneth cell development
2.36	0.017	*DEFA6*	Defensin Alpha 6—antimicrobial peptide
2.34	0.029	*LOC100134294*	Probe ILMN_3238960—unknown function; upregulated in ulcerative colitis
2.29	0.013	*M160*	CD163 Molecule Like—belongs to cysteine-rich scavenger receptors; may characterize a subset of CD163+colonic CD14+cells
2.28	0.021	*GCG*	Glucagon—the transcript encodes glucagon and three other enterohormones, including GLP-2 that stimulates crypt proliferation and gut barrier function
2.22	0.020	*CEACAM5*	CEA Cell Adhesion Molecule 5—cell adhesion molecule; may be elevated in various cancers
2.20	0.017	*SST*	Somatostatin—peptide hormone best known for inhibiting the pituitary; also interacts with enterohormones
2.20	0.007	*PDGFRA*	Platelet Derived Growth Factor Receptor Alpha; its activity associates with eosinophilia; *PDGFRA* rearrangement may cause chronic eosinophilic leukemia
2.19	0.017	*FCGBP*	Fc Fragment Of IgG Binding Protein (another probe)
2.17	0.020	*CD24*	CD24 Molecule—a peptide binding lectins; expressed by B-cells and granulocytes
2.17	0.038	*HLA-DQA1*	Major Histocompatibility Complex, Class II, DQ Alpha 1—presents antigens; its variants associate with autoimmunity against infliximab in inflammatory bowel diseases
2.13	0.043	*ITLN2*	Intelectin 2—may bind bacterial polysaccharides

**Table 4:** Top 10 transcripts underexpressed in ileal mucosa ranked by increasing absolute log_2_ fold change (logFC) in asthma vs controls. FDR—false-discovery ratio (Benjamini-Hochberg)

logFC	Average expression	p_FDR_	Gene	Gene name and function
− 1.93	6.94	0.028	*LOC440525*	Probe ILMN_1662409—unknown function; increased expression in the intestine after congenital heart defect surgery
− 1.92	7.86	0.017	*LOC731789*	Probe ILMN_3233239—unknown function; related to rs11015207 that associates with maximal oxygen uptake response after training
− 1.85	7.91	0.009	*PDE4A*	Phosphodiesterase 4A—regulates cellular cyclic adenosine monophosphate (cAMP); related to asthma; asthma drug target
− 1.84	9.00	0.017	*LOC100129201*	Probe ILMN_3263918—unknown function; might be hypertension-related
− 1.84	7.31	0.036	*PCK1*	Phosphoenolpyruvate Carboxykinase 1—controls gluconeogenesis; regulates the citric acid cycle
− 1.78	7.67	0.016	*TAF13*	TATA-Box Binding Protein Associated Factor 13—involved in initiation of transcription by RNA polymerase II; its mutations associate with schizophrenia
− 1.76	6.70	0.027	*LOC100130422*	Probe ILMN_3242709—function unknown
− 1.74	8.55	0.033	*HINT3*	Histidine Triad Nucleotide Binding Protein 3—may act on alpha-phosphate of ribonucleotides
− 1.74	7.13	0.017	*KREMEN2*	Kringle Containing Transmembrane Protein 2—by enhancing endocytosis of LRP5 and LRP6 inhibits signalling through Wnt/β-catenin pathway
− 1.71	7.90	0.017	*TAF13*	TATA-Box Binding Protein Associated Factor 13

Notably, no statistically significant differential gene expression was detected when comparing other asthma cells or biopsies with control samples, including CD4+, CD8+, CD14+, CD15+, CD19+ cells, platelets (low expression), transverse colon or rectum biopsy. Complete results of differential expression analyses are given in Additional file 1: Table S1.

Because of the influence of smoking on asthma phenotypes and CD, the analyses were repeated after the exclusion of smokers (n = 4, one with asthma and three controls). The reduction of sample size resulted in the loss of statistical significance (lowest FDR-adjusted *p* = 0.087). Therefore, only the top 5% of the most strongly differentially expressed genes were analyzed. They included 13 out of 20 of the most overexpressed genes from the whole-group analysis (Table 3), demonstrating that differences in the expression levels of *FCGBP, MUC2, OLFM4, C1QB, ITLN1* were present in non-smokers (Additional file 2: Table S2). Moreover, ranking by the highest log-fold change was similar. *FCGBP* was the most overexpressed transcript, followed by *DEFA1B, C1QB, CD24, MUC2*, and *ITLN1*. Likewise, the most downregulated genes were unaffected by the exclusion of smokers (i.e., *PDE4A, KREMEN2, TAF13*). Therefore, the key results of this study seem to be independent of smoking.

### Gene ontology

**Fig. 2:**
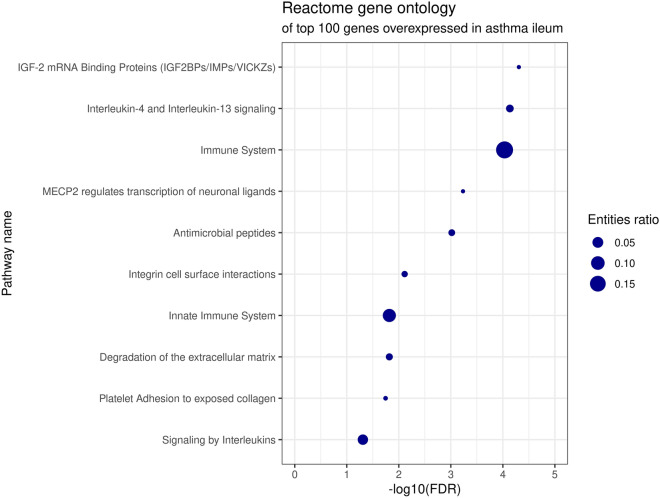
Reactome pathway enrichment of top 100 genes overexpressed in the ileum of patients with asthma vs controls. Entities ratio reflects the size of the pathway (e.g., “Immune System” contains 2713 genes, of a total of 14,721 genes recognized by Reactome, which yields 0.18)

**Fig. 3:**
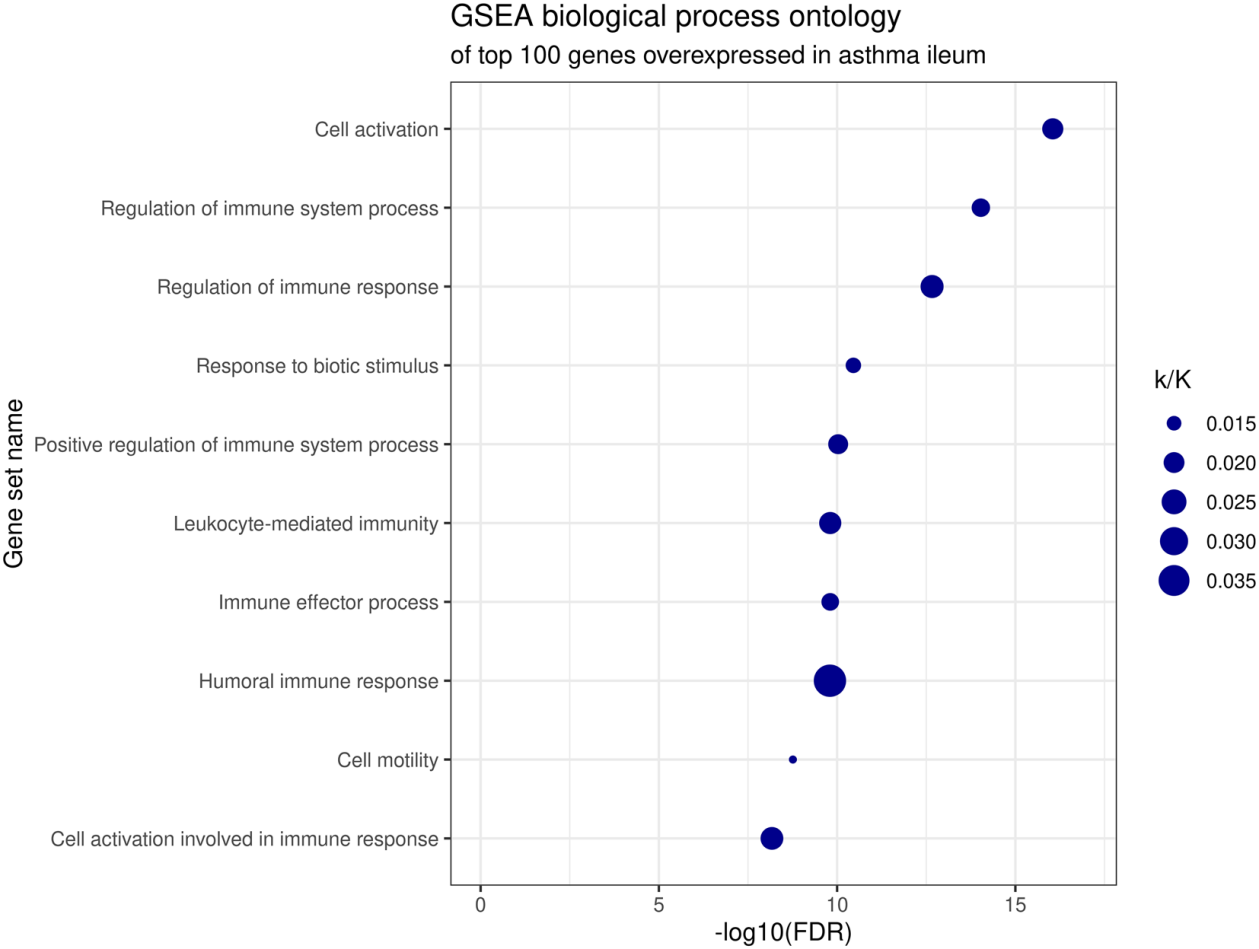
Biological process gene set enrichment of top 100 genes overexpressed in the ileum of patients with asthma vs controls. The ratio k/K reflects the proportion of overlapping genes to all genes in the specific set (e.g., 14 genes were in overlap with “humoral immune response”, of a total of 370 entities in this ontology set, yielding 0.038). Between 16 and 30 out of 100 investigated genes were in overlap with each of the individual listed pathways (Additional file 3: Table S3)

Gene ontology using Reactome and GSEA revealed pathway enrichment only for overexpressed transcripts. Reactome results included various themes, including insulin-like growth factor 2 mRNA-binding proteins, interleukin 4 and 13 signaling, and the immune system (general term), followed by antimicrobial peptides, integrins, and the extracellular matrix (Fig. 2). GSEA involve immune-related cellular activation, and responses to the microbiota (Fig. 3). The full results of the ontology analyses from Reactome and GSEA, including the involved genes, are presented in Additional file 3: Table S3.

### Study of overlap with CD

When examining CD and control data from Vancamelbeke et al., the number of ileal samples included is 78, consisting of 67 patients with CD and 11 controls. Out of 8895 unique genes (10,667 probes) from the asthma dataset, 6,850 were also present in the dataset by Vancamelbeke et al. The two datasets were intersected to include only those genes present in both datasets. Of these, 1,033 were over- and 1,293 underexpressed in CD compared with the controls. The overlap of genes that are overexpressed in asthma and CD is illustrated in Table 5. As much as 34% of unique genes that are overexpressed in asthma, are also overexpressed in CD ileum (Table 5), with concordance between those genes overexpressed in asthma and CD (p = 3.66 × 10^–7^). Intersection of the top 150 over- and underexpressed genes is presented in Table 6. The main similarities included overexpression of *STAT1* and underexpression of *RARA*. However, only *NELF* can be found within 241 loci that were examined in IBD patients by genome-wide association studies (de Lange et al. [18]), notably in an region of dense immune gene clustering on chromosome 6 that also contains *HLA* and *TNF* genes (top SNP rs6927022). Reactome ontology of genes that are overexpressed in asthma and CD linked them to the unfolded protein response (FDR = 1.00 × 10^–5^), activation of chaperone genes (FDR = 2.06 × 10^–5^), interleukin-6 (FDR = 0.008), and interferon signaling (FDR = 0.013).

**Table 5:** Matrix illustrating overlap between genes overexpressed in asthma (CEDAR cohort) vs Crohn’s disease (Vancamelbeke et al.) in comparison with respective controls

Group	Asthma, overexpressed, n = 848 unique genes	Asthma, not overexpressed, n = 6002 unique genes
CD, overexpressed, n = 1033	286	747
CD, not overexpressed, n = 5817	562	5255

McNemar’s test was used since each gene was assessed twice (overexpression in asthma, in CD): p = 3.66 × 10^–7^

**Table 6:** Examples of genes most strongly over- or under-expressed in asthma and Crohn’s disease

Group	Asthma overexpressed	Asthma underexpressed
CD overexpressed	*ATP2C1, BTN3A2, CCND2, CLINT1, LPIN1, MELK, MXRA5, PDIA5, SAMD9L, STAT1, XBP1, ZNFX1*	*CBX3, DLEU2, SIL1*
CD underexpressed	None	*ALDOC, CBS, CRIP1, DUSP3, FAM134A, FOXO4, HRH1, JDP2, MKNK2, NELF, PIGS, RARA, RHOBTB2*

Top 150 over- or underexpressed genes were intersected

## Discussion

Intestinal transcriptomes in asthma are of interest because of the involvement of microbiota and environmental exposures in disease pathogenesis. However, they have not been investigated to date, which presents a gap in our understanding that this study attempts to address. First, we demonstrated that asthma-specific changes in the ileum persist even in the absence of differences in various leukocyte subtypes. Secondly, we identified a degree of similarity between the ileal transcriptomes in asthma and CD, with some transcripts similarly affected by both conditions. Thus, the study exposed the small intestine as an important organ for future asthma research focusing on discerning between cause and effect, with potential therapeutic implications.

### IgGFc-binding protein—the most overexpressed gene in asthma ileum

The most overexpressed gene, *FCGBP*, encodes IgGFc-binding protein, is mostly expressed in the intestine and resembles mucins not only by localization of the mature protein in the mucus, but also when its structure is analyzed [19, 20]. FCGBP is known to be produced by goblet cells [21], which covalently bind MUC2, contributing to the mucin mesh [22]. FCGBP allows trefoil factor 3 (TFF3) to attach to the network, providing a reservoir from which TFF3 can be cleaved [23], possibly to aid mucosal healing. Interestingly, *FCGBP* is overexpressed in the intestinal crypts of ulcerative colitis patients [24]. It also associates with endodermal organ progenitors [25] and is differentially expressed in colon cancer [26]. Circulating FCGBP associated with systemic sclerosis was speculated to arise from intestinal goblet cells [27].

### Mucin 2

We found that *MUC2* (mucin 2), the product of which binds FCGBP, was the second most overexpressed gene. Overexpression of mucins in the airways is one of the characteristic features of asthma, although this has not been sufficiently characterized in the intestine. A polymorphism in *MUC2* was linked to asthma, but because MUC2 is not a major airway mucin it was speculated that the variant may be related to a haplotype encompassing airway-related *MUC5AC* [28]. In fact, MUC2 in humans is characteristic of intestinal goblet cells [29]. It can also be upregulated in Paneth cells by IL-13 through IL-9 expression [30]. In contrast, a murine model has previously revealed that IL-4 is able to stimulate the expression of *MUC5AC* and not *MUC2* [31]. *MUC2* can also be induced by leukotrienes [32], and its production may be reduced by pranlukast, a leukotriene receptor antagonist [33]. In pediatric CD mucosa, the expression of *MUC2* is inversely associated with IL-8, which promotes granulation tissue formation [34]. The overexpression of *MUC2* in the context of the asthmatic ileum underscores the possibility of its expression being induced in Paneth cells through IL-13 expression.

### Paneth and goblet cell activation or overgrowth are implicated in the asthmatic ileum

Increased expression of intelectin 1, and mucin 2 may implicate both Paneth and goblet cells (according to PanglaoDB). However, overexpression of defensins, lysozyme, *PLA2G2A*, and *REG4* suggests overactivity and/or overgrowth of Paneth cells. Although Paneth cells are susceptible to IL-4 expression, its presence would reduce than increase lysozyme activity [35]. Conversely, IL-13 can promote degranulation of Paneth cells and the production of antimicrobial peptides [36]. Interestingly, *FCGBP* can not only be expressed by Paneth and goblet cells, but also enterocytes. Expression of the cell surface protein product of *CEACAM5* would then suggest involvement of goblet cells. Furthermore, *SST* and *GCG* might implicate enteroendocrine cells and *OLFM4* could point towards stem cells or immature enterocytes, as well as Paneth or crypt cells. Taken as a whole, these results potentially demonstrate stimulation of Paneth cells, likely due to IL-13 expression.

### Low correspondence with expression profiles in asthma airways

A large integrative study of asthma transcriptomics in various tissues [37] revealed organism-wide changes in IL-1β and ERK signaling. Airway samples in asthma have dysregulated TGFβ, as well as IL-13. Interestingly, machine learning (support vector machines) showed that differences between asthma and control samples were more discriminatory in macrophages than in the epithelial cells. We found little overlap between the genes that were overexpressed to the highest degree in the ileum of asthma patients, and asthma-specific genes identified across tissues by Ghosh et al. [37]. However, a transcriptomic study of airway epithelial cells by Kicic et al. revealed that the response to a biotic stimulus was the top upregulated pathway in asthma [38], and this theme was also enriched in the ileum. Of the key genes that are overexpressed in the ileum, only *OLFM4* was also found to be overexpressed in the asthmatic airway [38]. OLFM4 has antiapoptotic properties, potentially promotes STAT4 activation and cellular adhesion, and has been considered a marker of intestinal stem cells [39]. It was recently found to be overexpressed in crypts from patients with ulcerative colitis and primary sclerosing cholangitis [40]. The overexpression of *OLFM4* in both intestinal and airway mucosa points towards a different source, rather than intestinal cells. A potential candidate could be neutrophils, which release OLFM4 during the formation of neutrophil extracellular traps (NETs) [41]. OLFM4 also seems to have specific functions in the extracellular matrix, which are not yet understood. *C1QB* and *HLA-DQA1* were overexpressed in the intestine, but were found to be reduced in asthma airways. Interestingly, *PDE4A*, encoding one phosphodiesterases targeted by asthma medication [42], was down-regulated in the ileum of patients with asthma. To summarize, there are very few similarities in asthma-related changes in the intestinal and airway epithelium. Increased response to a biotic stimulus may be considered one common denominator that may involve *OLFM4*-expressing neutrophils.

By assuming that these specific changes in the ileum are primarily linked with, or intertwined with airway inflammation in asthma, a mechanistic link between the two may be found through innate immunity. If the intestine plays an important role in triggering and maintaining innate systemic immunity, then hyperactivation followed by interventions in the gut should be an efficacious treatment for asthma. The evidence regarding the impact of probiotics on asthma development is mixed but may prevent wheezing or even the disease itself (7,8). Moreover, intestinal inflammation precedes clinically overt asthma, supporting this assumption (9). The microbiota, short-chain fatty acid production, autophagy, and the parasympathetic tone (10) are other asthma-related gut factors that require further attention. Our data support the notion that asthma may be susceptible to reduction of immune activation in the gut via nutritional, lifestyle, probiotic, and pharmacological interventions.

### Parallels with Crohn’s disease

A major difficulty arising in the analysis of CD transcriptomes is discriminating between changes that are causative of inflammation and other alterations, which help mitigate its effects. Are genes that are over- or under-expressed in both the asthma and CD ileum typical of inflammation? Gene ontology implicated IL-6 (*STAT1*), interferon-related genes (*STAT1, ZNFX1*), and unfolded protein response (*XBP1, PDIA5*), all of which may be already downregulated in CD. A defective unfolded protein response (i.e., due to XBP1 deficiency) has been demonstrated previously to stimulate intestinal inflammation [43]. In goblet cells exposed to interferon-ϒ, *MUC2* transcription is upregulated (as in this study), but this does not result in increased production of the MUC2 protein [44]. Interestingly, in our analysis, we did not observe an increase in the marker of endoplasmic reticulum stress *GRP78* (*HSPA5*) [45]. In summary, the similarity of the asthmatic ileum expression profile to CD may stem from endoplasmic reticulum stress and/or inflammation. However, other genes are also involved, the functions of which are under researched.

### Research directions

Although this research is of a basic scientific nature, it yields new clinical hypotheses and directions. First, considering blood concentration of FCGBP is elevated in autoimmune diseases [27], its assessment may be correlated with intestinal expression in asthmatics. Second, since asthma-specific intestinal involvement seems to exist, fecal calprotectin and other biomarkers of intestinal inflammation and tight junction proteins could be explored. It is worth noting that fecal calprotectin is a relatively inexpensive and non-invasive assessment that, despite being a clinical-grade examination, remains underexplored in asthma. Of note is that serum calprotectin concentration in asthma patients correlated weakly but significantly with forced expiratory volume in one second (FEV1%) [46]. Third, additional studies of the ileum and colon in asthmatic patients can be carried out within cancer screening programs to uncover asthma subset-specific changes and correlate co-expression modules with disease characteristics. Finally, if transcriptomics are not available, endoplasmic reticulum stress alone can be measured in the ileum using the expression of *GRP78* (*HSPA5*) [45].

### Generalizability

This study is limited by the lack of phenotypic characterization of asthma (i.e., subtypes, spirometry, treatment) and a small sample size, which restricts its generalizability but provides sufficient evidence to demonstrate important differences in asthmatic patients when compared to their respective controls. A major advantage of the study, which stems from the scale of the CEDAR cohort, is close matching between asthmatic and control participants. Moreover, this work focuses on a transcriptomic analysis of microarray data, the results from which could potentially benefit from a confirmation with polymerase chain reaction or protein-based assays. It should also be kept in mind that expression levels often do not correlate with protein concentrations. Nevertheless, the presented results are sufficient to draw relevant conclusions and attract attention to the ileum of asthmatic patients.

## Conclusion

In summary, the ileal expression profile in asthmatic patients revealed an up-regulation of genes involved in the innate (*FCGBP* and mucin 2, defensins, intelectins, lysozyme) and adaptive immune systems (IL-4 and IL-13 pathways), highlighting possible involvement of Paneth and goblet cells. Therefore, the ileum harbors asthma-specific transcriptomic changes and further studies of the small intestine in asthma are warranted, including protein profiling and single-cell RNA sequencing.

## Supplementary Information

**Additional file 1: Table S1.** Full results of differential expression analysis.

**Additional file 2: Table S2.** Differential expression analysis of the ileum in non-smoking patients with asthma vs. non-smoking controls.

**Additional file 3: Table S3.** Results of gene ontology analyses.

## Data Availability

The datasets are publicly available: CEDAR at the Array Express (E-MTAB-6667) and the study by Vancamelbeke et al. at the Gene Expression Omnibus (GSE75214).

## Abbreviations

BMI: Body mass index
CEDAR: Correlated Expression and Disease Association Research
CD: Crohn’s disease
FCGBP: IgGFc-binding protein
IBD: Inflammatory bowel diseases
MUC2: Mucin 2
